# Risk of stroke in cancer survivors using a propensity score-matched cohort analysis

**DOI:** 10.1038/s41598-021-83368-w

**Published:** 2021-03-10

**Authors:** Eiko Saito, Manami Inoue, Norie Sawada, Yoshihiro Kokubo, Kazumasa Yamagishi, Hiroyasu Iso, Taichi Shimazu, Taiki Yamaji, Motoki Iwasaki, Shoichiro Tsugane, JPHC Study Group

**Affiliations:** 1 Division of Cancer Statistics Integration, Center for Cancer Control and Information Services, National Cancer Center, 5-1-1 Tsukiji, Chuo-ku, Tokyo, 104-0045 Japan; 2 Epidemiology and Prevention Group, Center for Public Health Sciences, Research Center for Cancer Prevention and Screening, National Cancer Center, 5-1-1 Tsukiji, Chuo-ku, Tokyo, 104-0045 Japan; 3 Department of Preventive Cardiology, National Cerebral and Cardiovascular Center, 6-1 Kishibe-Shinmachi, Suita, Osaka 564-8565 Japan; 4 Department of Public Health Medicine, Faculty of Medicine, and Health Services Research and Development Center, University of Tsukuba, 1-1-1 Tennodai, Tsukuba, Ibaraki 305-8575 Japan; 5 Public Health, Department of Social and Environmental Medicine, Osaka University Graduate School of Medicine, Suita, Osaka 565-0871 Japan

**Keywords:** Cancer, Oncology

## Abstract

Little is known about the risk of cerebrovascular disease in cancer survivors. We aimed to assess the association between incident cancer and the subsequent risk of stroke using a large-scale, population-based prospective study. 74,530 Japanese aged between 40 and 69 years at baseline study were matched by the status of cancer diagnosis during follow-up using propensity score nearest-neighbor matching with allowance for replacement. A total of 2242 strokes were reported during 557,885 person-years of follow-up. Associations between incident cancer and the subsequent risk of all strokes, cerebral infarction, and intracerebral hemorrhage were assessed using a Cox proportional hazards model stratified on the propensity score-matched pairs. No significant association was observed between the status of cancer diagnosis of all types, gastric, colorectal and lung cancer, and subsequent occurrence of all strokes, cerebral infarction, and intracerebral hemorrhage. However, analysis by discrete time periods suggested an elevated risk in cancer patients for one to three months after a cancer diagnosis in all stroke (HR, 2.24; 95% CI, 1.06, 4.74) and cerebral infarction (HR, 2.62; 95% CI, 1.05, 6.53). This prospective cohort study found no association between the status of cancer diagnosis and the subsequent occurrence of all strokes and its subtypes during the entire follow-up period but suggested an increase in stroke risk during the active phase of malignancy.

## Introduction

A growing number of studies have reported an elevated risk of stroke in cancer survivors. A study in the US showed that patients with cancers of all types, breast, lung, pancreas, colorectum, and prostate had an elevated risk of stroke^1,2^. In Asian populations, studies in Taiwan reported a higher risk of stroke in patients with cervical cancer^3^ and head and neck cancer^4^. Previous studies indicate that patients with mucinous adenocarcinoma in the pancreas, lung, and gastrointestinal tract have an increased incidence of thromboembolic deaths^5^. The hypothesis was that stroke risk is increased in cancer patients due to the activity of tumor cells that activate the coagulation system^5,6^, or to the effect of oncological treatments^6–8^.

However, the majority of such reports derive from patient studies or health insurance claims data, and only a few large-scale prospective studies have investigated the risk of cerebrovascular disease in cancer survivors from a general population^1,4^. Even among the existing studies, the majority followed a retrospective matched cohort design, and the underlying lifestyle factors were poorly adjusted due to the unavailability of information, which may have lead to biased estimates. As for the case of Japan, one study with a prospective cohort design reported the risk of stroke in a Japanese community; sample size in this study was only around 3500 subjects^9^, however, and the number of both cancer and stroke cases was limited, preventing substantial analysis.

In the context of global population aging, the burden of cancer is expected to increase further. Investigating the magnitude of cardiovascular complications associated with cancer will help identify prevention strategies for cardiovascular diseases in cancer survivors and eventually improve the prognosis and survival of these patients. Here, we used large-scale, population-based prospective cohort data in a general population with systematic cancer and stroke registration systems to investigate the risk of stroke after a cancer diagnosis.

## Methods

### Study population

**Figure 1:**
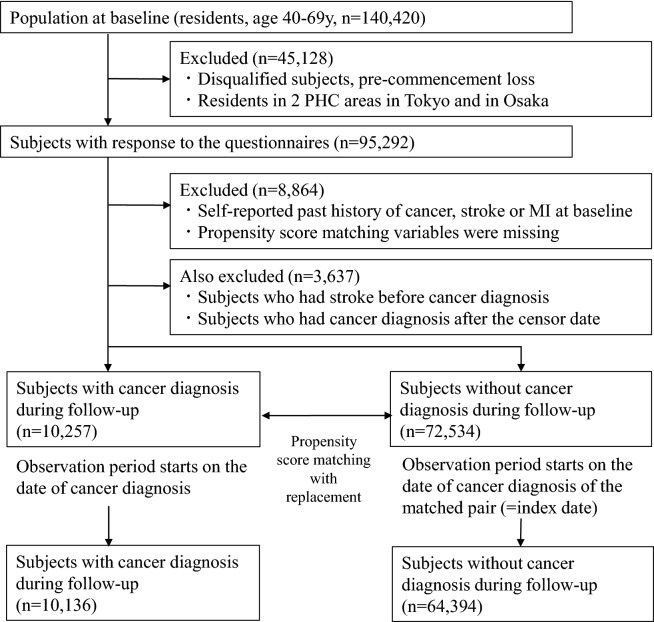
Flowchart of the study participants.

Details of the Japan Public Health Center-based Prospective Study have been described elsewhere^10^. The baseline study for Cohort I started in 1990 and that for Cohort II in 1993, covering a total of 140,420 participants in 11 public health center areas. The study enrolled participants aged 40 to 59 years in Cohort I and 40 to 69 years in Cohort II. Figure 1 shows a flowchart of participants included in the present study. Two PHC areas in metropolitan Tokyo (n = 7097) and Osaka (n = 16,427) were excluded due to a lack of complete data on cancer and stroke incidence. Of all participants, those with non-Japanese nationality, incorrect birth date, multiple registration, or pre-commencement loss were excluded due to non-eligibility (n = 309). Of eligible participants, 95,292 subjects (45,404 men and 49,888 women) or 81.7% of the subjects completed the questionnaire, which included demographics, anthropometric information, smoking, alcohol intake, physical exercise, and dietary habits. We excluded participants with self-reported histories of cancer, stroke, or myocardial infarction (n = 3648) at baseline to avoid bias induced by pre-existing medical conditions. We further excluded those without information on anthropometrics, smoking status, alcohol intake, regular exercise, history of diabetes or hypertension, and taking of a health examination (n = 5216). To avoid reverse causality between stroke and cancer, we also excluded participants who were diagnosed with cancer after stroke diagnosis (n = 495). To avoid preclinical cases of cancer-causing bias, we also excluded subjects with a confirmed diagnosis of cancer after the censor date (n = 3142). Finally, 82,791 participants were included (38,728 men and 44,063 women). The study was approved by the Institutional Review Boards of the National Cancer Center in Tokyo, Japan (approval number: 2001-021), with reference to relevant ethical guidelines for medical research in Japan. Informed consent was obtained from each participant implicitly when they completed the baseline questionnaire, in which the purpose of the study and follow-up methods were fully described and explained. Detailed information on the study was mailed to each participant and is published on the study website (http://epi.ncc.go.jp/jphc).

### Assessment of exposure

Our study used incident cancer that occurred during the follow-up period before the diagnosis of stroke or the censor date as an exposure variable. We identified cancer cases of all sites, stomach, colorectum, and lung, through active patient notification from major local hospitals and linkage with data from population-based cancer registries^11^. Cancer diagnoses were coded with the Third Edition of the International Classification of Diseases for Oncology for stomach (C16.0–16.9), colorectum (C18.0–20.9), and lung (C34.0–34.9), respectively. In cases where a participant had multiple incidences of cancer, only the first incidence was used. We classified subjects without a cancer diagnosis during follow-up as the reference group for comparison against subjects with a cancer diagnosis of any type, gastric cancer, colorectal cancer, and lung cancer. We also classified clinical stage at cancer diagnosis according to the SEER Summary Staging Manual 2000, namely "Localized (localized to the tissue of origin)," "Regional (spread to an adjacent organ, muscle, connective tissue, fat, serosa or regional lymph node)," and "Distant (spread to another place in the body)"^12^.

### Ascertainment of stroke cases

The endpoints of this study were the incidences of total stroke, cerebral infarction, and intracerebral hemorrhage. Subarachnoid hemorrhage was not included. In most cases, physicians in the 81 major hospitals registered within the JPHC study area are blinded to patient lifestyle information and reviewed the medical records. Stroke cases were confirmed according to the National Survey of Stroke criteria^13^, which requires a constellation of a neurological deficit of sudden or rapid onset lasting at least 24 h or until death. A diagnosis of intracerebral hemorrhage and cerebral infarction was determined through computed tomography scans, magnetic resonance images, or autopsy. Details of confirmation procedures have been described elsewhere^14^.

### Follow-up

Study participants who experienced incident cancer during the follow-up period entered into the study at the time of cancer diagnosis and were followed until the censor date—date of diagnosis of stroke, date of death, date of migration out of the study area, or the end of follow-up (31 December 2009)—whichever came first. If the date of cancer diagnosis was the same as the date of death (e.g., cases in which cancer occurrence was reported from the death certificate only), these participants were excluded. Study participants without a cancer diagnosis entered into study on the date of cancer diagnosis of their propensity-score matched pair and were followed until the censor date.

### Statistical analysis

For the propensity score-matched cohort analyses, we used subjects with a cancer diagnosis during follow-up (n = 10,257) and subjects without a cancer diagnosis during follow-up (n = 72,534). We calculated the propensity score using a multivariate logistic regression model with the possible confounding factors of gender; age at baseline; public health center area; smoking status (never, former, < 20 cigarettes/day, and ≥ 20 cigarettes/day); BMI (in kg/m^2^; < 18.5, 18.5–< 25, 25–< 30, and ≥ 30); alcohol intake (never/former, < 1 time/week, regular (ethanol converted g/d) [< 23, 23–< 46, 46–< 69, 69–< 92, and ≥ 92]); leisure-time sports or physical exercise (< almost daily, almost daily); histories of hypertension and diabetes (yes or no); and medical examination history within the past year (yes or no). Propensity scores were used to match cancer survivors to the subjects without cancer diagnosis during follow-up according to nearest-neighbor matching with sample replacement in the cancer survivor group to allow for retention of the full sample size. Following this process, control subjects were assigned an index date (i.e., starting date of the observation period), corresponding to the date of cancer diagnosis of their matched cancer patient. If the censor date of the matched control occurred before the index date, these subjects were excluded from the analysis (n = 8261).

Cox proportional hazards regression models stratified on the propensity score-matched pairs were used to assess the associations between incident cancer and subsequent risk of stroke. The hazard ratios (HRs) and 95% confidence intervals (95% CI) were then estimated^15^. Further, we analyzed the associations between any cancer and stroke by discrete time periods after diagnosis of cancer, namely by 0 to < 1 month, 1 to < 3 months, 3 to < 6 months, and 6 to < 12 months. In our secondary analysis, we assessed the associations between clinical stage at cancer diagnosis and subsequent risk of stroke. Also, to assess the robustness of our analyses, we compared the results obtained from propensity score matching with replacement with those obtained from one-to-one propensity score matching without replacement. We conducted tests for non-proportionality in the estimated hazards by Therneau and Grambsch to evaluate departures from the proportional hazards assumption, and no violation of the assumption was reported. All *p* values were two-sided, with values smaller than 0.05 indicating statistical significance. All analyses were conducted with STATA version 14.0 software (StataCorp LP).

## Results

Table 1 summarizes the baseline characteristics of study participants by the status of cancer diagnosis during follow-up. At the time of study recruitment (1990–1994), participants with cancer during the follow-up period were older than those without a cancer diagnosis. Supplemental Table 1 compares participants who responded to the study questionnaires by their inclusion status in the final analysis. Participants included in the final analysis had a lower prevalence of hypertension and diabetes and tended to be younger than excluded participants. Subjects with incident cancer cases were also more likely to smoke relative to those without a cancer diagnosis. During 557,885 person-years of follow-up, we observed 2242 strokes after propensity score matching with replacement. Of these, 1421 cases were cerebral infarction, 594 were intracerebral hemorrhage, 217 were subarachnoid hemorrhage, and 10 were unknown cases. Hazard ratios with 95% CIs for the association between incident cancer of all types and by cancer site during follow-up and the subsequent risk of stroke are presented in Table 2. No significant association was seen between the status of cancer diagnosis of all types, gastric cancer, colorectal cancer, and lung cancer and subsequent occurrence of all strokes, and cerebral infarction and intracerebral hemorrhage. The results were concordant with those obtained from one-to-one propensity score matching without replacement (Supplemental Table 2). However, our analysis by discrete time periods showed a significantly elevated risk of all strokes from one to three months after a cancer diagnosis in all stroke (HR, 2.24; 95% CI, 1.06, 4.74) and cerebral infarction (HR, 2.62; 95% CI, 1.05, 6.53) (Table 3). In addition, analysis by clinical stage at diagnosis for gastric cancer showed a significantly elevated risk of all strokes (HR, 4.60; 95% CI, 1.73, 12.24) and cerebral infarction (HR, 6.17; 95% CI, 2.21–17.27) in subjects who were diagnosed with distant metastatic gastric cancer prior to the stroke (Supplemental Table 3).

**Table 1:** Baseline characteristics of participants by propensity score-matched pair.

Characteristics	All cancer			Gastric cancer^3^			Colorectal cancer^4^			Lung cancer^5^		
	Subjects with incident cancer	Matched pair	*p* value^1^	Subjects with incident cancer	Matched pair	*p* value^1^	Subjects with incident cancer	Matched pair	*p* value^1^	Subjects with incident cancer	Matched pair	*p* value^1^
**Total participants (n = 74,530)**	10,136	64,394		1954	64,685		1948	64,245		1173	63,424	
Female (%)	39.3	56.8	< 0.001	29.6	56.5	< 0.001	39.3	56.7	< 0.001	29.1	56.8	< 0.001
Age at baseline (years), mean ± SE^2^	54.9 ± 0.08	51.1 ± 0.03	0.011	55.2 ± 0.17	51.1 ± 0.03	0.009	54.5 ± 0.17	51.1 ± 0.03	0.083	56.5 ± 0.21	51.1 ± 0.03	0.006
Body mass index (kg/m^2), mean ± SE	23.5 ± 0.03	23.6 ± 0.01	0.444	23.2 ± 0.07	23.6 ± 0.01	0.502	23.8 ± 0.07	23.6 ± 0.01	0.179	23.1 ± 0.09	23.6 ± 0.01	0.810
Current smoker (%)	36.7	24.8	< 0.001	41.6	25.0	< 0.001	34.3	24.8	< 0.001	58.0	24.8	< 0.001
Current drinker (%)	53.3	45.6	< 0.001	62.0	45.8	< 0.001	54.6	45.6	< 0.001	53.5	45.6	< 0.001
Sports or physical exercise almost daily (%)	5.5	4.7	< 0.001	4.5	4.7	0.585	5.8	4.7	0.030	5.3	4.7	0.350
History of hypertension (%)	20.4	15.3	< 0.001	20.4	15.3	< 0.001	22.7	15.3	< 0.001	18.7	15.3	0.001
History of diabetes (%)	6.0	3.8	< 0.001	6.7	3.9	< 0.001	5.8	3.9	< 0.001	5.3	3.8	0.009
Attended health examination or screening within the past 1 year (%)	79.8	78.6	< 0.001	79.8	78.7	0.209	80.2	78.6	0.097	77.7	78.7	0.397

^1^ANOVA or chi-square-test.

^2^*SE* standard error.

^3^Excluding subjects with incident cancer during follow-up other than gastric cancer.

^4^Excluding subjects with incident cancer during follow-up other than colorectal cancer.

^5^Excluding subjects with incident cancer during follow-up other than lung cancer.

**Table 2:** Hazard ratios of stroke during follow-up by stroke type.

	All stroke			Cerebral infarction			Intracerebral hemorrhage		
	Subjects without cancer	Cancer survivors	95% CI	Subjects without cancer	Cancer survivors	95% CI	Subjects without cancer	Cancer survivors	95% CI
Person-years (n = 557,885)									
All Cancer									
Number of stroke cases	1963	279		1227	194		535	59	
Adjusted HRs^1^	1.00	0.93	(0.78–1.11)	1.00	0.91	(0.73–1.12)	1.00	0.96	(0.66–1.41)
Gastric Cancer^2^									
Number of stroke cases	2056	79		1262	54		589	17	
Adjusted HRs^1^	1.00	1.19	(0.92–1.53)	1.00	1.19	(0.88–1.62)	1.00	1.10	(0.65–1.85)
Colorectal Cancer^3^									
Number of stroke cases	1975	77		1235	59		546	12	
Adjusted HRs^1^	1.00	1.15	(0.90–1.47)	1.00	1.32	(1.00–1.75)	1.00	0.65	(0.36–1.21)
Lung cancer^4^									
Number of stroke cases	1737	25		1096	19		487	5	
Adjusted HRs^1^	1.00	1.01	(0.64–1.60)	1.00	1.07	(0.63–1.83)	1.00	1.08	(0.43–2.70)

^1^The number of stroke cases in this table was calculated after propensity score matching with replacement, in which propensity scores were predicted by age, gender, PHC area, smoking status (never, former, < 20 cigarettes/day, ≥ 20 cigarettes/day), BMI (< 18.5, 18.5–< 25, 25–< 30, 30+), alcohol intake (never/former, < 1 time/w, regular (g/d) [< 23, 23–< 46, 46–< 69, 69–< 92, 92+] ), leisure-time sports or physical exercise (< almost daily, almost daily), history of diabetes or hypertension, and undergoing a health examination (no, yes).

^2^Excluding subjects with incident cancer during follow-up other than gastric cancer.

^3^Excluding subjects with incident cancer during follow-up other than colorectal cancer.

^4^Excluding subjects with incident cancer during follow-up other than lung cancer.

**Table 3:** Hazard ratios of stroke during discrete time periods after diagnosis of cancer.

Time after cancer diagnosis	All stroke			Cerebral infarction		
	Subjects without cancer	Cancer survivors	95% CI	Subjects without cancer	Cancer survivors	95% CI
**0**–**< 1 month**						
Number of stroke cases	20	9		14	8	
Adjusted HRs^1^	1.00	1.51	(0.59–3.87)	1.00	1.63	(0.58–4.59)
**1**–**< 3 month**						
Number of stroke cases	34	16		19	12	
Adjusted HRs	1.00	**2.24**	**(1.06–4.74)**	1.00	**2.62**	**(1.05–6.53)**
**3**–**< 6 month**						
Number of stroke cases	66	11		45	8	
Adjusted HRs^1^	1.00	0.72	(0.32–1.66)	1.00	0.67	(0.25–1.82)
**6**–**< 12 month**						
Number of stroke cases	107	21		60	13	
Adjusted HRs^1^	1.00	0.90	(0.46–1.76)	1.00	0.91	(0.40–2.05)

^1^The number of stroke cases in this table was calculated after propensity score matching with replacement, in which propensity scores were predicted by age, gender, PHC area, smoking status (never, former, < 20 cigarettes/day, ≥ 20 cigarettes/day), BMI (< 18.5, 18.5–< 25, 25–< 30, 30+), alcohol intake (never/former, < 1 time/w, regular (g/d) [< 23, 23–< 46, 46–< 69, 69–< 92, 92+] ), leisure-time sports or physical exercise (< almost daily, almost daily), history of diabetes or hypertension, and undergoing a health examination (yes, no). Numbers marked in bold indicate numbers that are significant on the 95% confidence limit.

## Discussion

This study is the first of its kind to investigate the association between incident cancer and the subsequent risk of stroke using a large-scale prospective study in a general population, with robust adjustment for lifestyle-related risk factors by propensity score matching. Our results from a Japanese population aged 40–69 years at baseline showed no elevation in the risk of stroke in subjects with cancer of any site compared to participants without a cancer diagnosis during follow-up. However, our analysis by discrete time periods after cancer diagnosis showed an increased risk of all strokes and cerebral infarction from one to three months after diagnosis. Further, additional analysis by clinical stage at cancer diagnosis suggested an elevated risk of cerebral infarction in survivors of distant gastric cancer. These results should be interpreted with caution since reproducibility may be limited due to a small number of cases.

Our results indicating an increased risk of stroke in cancer survivors in the first three months corroborate with the findings from the US^2^. One possible reason for the higher stroke risk in cancer patients may be oncological treatment, since our results showed a significantly higher risk of stroke from one to three months after a cancer diagnosis, which predominantly corresponds with the initial phase of cancer treatment. Surgery, the first choice for advanced cancer at the time of the observation period, is associated with postoperative immobility and increased hemostatic system function^16^. Some of the platinum-based compounds and angiogenesis inhibitors used for the treatment of cancer have also been associated with increased thrombotic risk^17^. In the case of gastric cancer, conventional chemotherapies such as cisplatin are known to cause arterial thrombosis with cerebrovascular ischemia^18^.

Further, the suggested association may be explained by the fact that those who experience cancer are more likely to possess shared cardiovascular risk factors. As for the case of gastric cancer, the shared risk factor is known to be smoking^19,20^. Smoking increases the probability of developing cancer at a younger age, which may explain the early onset of cancer before the occurrence of stroke^21^. Besides lifestyle-related risk factors such as smoking, the hypercoagulability of tumor cells located in the gastrointestinal tract has also been reported^6^. Potential mechanisms include increased production of tissue factor and tumor pro-coagulant; secretion of inflammatory cytokines, including vascular endothelial growth factor; tumor-cell adhesion; and activation of the endothelium^22,23^. Such hypercoagulability might also cause thrombosis in the cerebral vasculature. Also, posttraumatic stress disorder (PTSD) after cancer diagnosis and treatment has been widely reported^24^, and studies from Taiwan and the US have shown that PTSD is associated with a heightened risk of any stroke and cerebral infarction in later life^25,26^. These factors, either combined or alone, may act to increase the risk of stroke, although the results should be interpreted with caution due to the limited number of cases.

This study has several limitations. First, the thrombogenic effects of chemotherapy or molecularly-targeted therapy vary by the type and combination of drugs used for oncological treatment. Because we did not have information on treatment after a cancer diagnosis, we were not able to conduct a more detailed analysis of the associations between cancer treatment and subsequent stroke events. Second, we were not able to examine the association of other cancer sites due to the limited number of exposure and outcome cases. Third, although the prospective nature of our study allowed robust adjustment of confounders, we were not able to perform further stratified analyses after matching the pairs by propensity scores that synthesized the confounders. Fourth, we were not able to assess the impact of lifestyle changes after a cancer diagnosis. Allowing for these limitations, this is the first large-scale prospective study from a general population with a confirmed diagnosis and date of diagnosis of both cancer and stroke. Because our analysis provides robust evidence, with adjustment for lifestyle risk factors, the presence of comorbidities, and cancer screening status, the chance of confounding is minimal.

In conclusion, this prospective cohort study found no association between cancer diagnosis status for all types, gastric cancer, and lung cancer and subsequent occurrence of all stroke, cerebral infarction, and intracerebral hemorrhage during the entire follow-up period, but suggested an increase in stroke risk during the active phase of malignancy. Future studies should aim to link the cohort data with medical records to assess the impact of different types of oncological treatments on subsequent cardiovascular events.

## Supplementary information

Supplementary information.

## Data Availability

For information on how to submit an application for gaining access to JPHC data/or biospecimens, please follow the instructions at http://epi.ncc.go.jp/en/jphc/805/8155.html.

## Abbreviation

JPHC study: The Japan Public Health Center-based Prospective Study
