# Supplementary material for: Risk of stroke in cancer survivors using a propensity score-matched cohort analysis
Source: Sci Rep. 2021 Mar 10;11:5599. doi: 10.1038/s41598-021-83368-w (PMC7946896; doi:10.1038/s41598-021-83368-w)
Supplement: Supplementary file 1 — Supplementary information. [file 41598_2021_83368_MOESM1_ESM.pdf]

Title: ***Risk of stroke in cancer survivors using a propensity score-matched cohort analysis.***

Eiko Saito[1]; Manami Inoue[2*]; Norie Sawada[2]; Yoshihiro Kokubo[3]; Kazumasa Yamagishi[4]; Hiroyasu Iso[5;] Taichi Shimazu[2]; Taiki Yamaji[2]; Motoki Iwasaki[2]; and Shoichiro Tsugane[2**]; for the JPHC Study Group**

1. Division of Cancer Statistics Integration, Center for Cancer Control and Information Services, National Cancer Center, 5-1-1 Tsukiji, Chuo-ku, Tokyo 104-0045 Japan
2. Epidemiology and Prevention Group, Center for Public Health Sciences, National Cancer Center, 5-1-1 Tsukiji, Chuo-ku, Tokyo 104-0045 Japan
3. Department of Preventive Cardiology, National Cerebral and Cardiovascular Center, 6-1 Kishibe-Shinmachi, Suita, Osaka, 564-8565 Japan
4. Department of Public Health Medicine, Faculty of Medicine, and Health Services Research and Development Center, University of Tsukuba, 1-1-1 Tennodai, Tsukuba, Ibaraki, 305-8575 Japan
5. Public Health, Department of Social and Environmental Medicine, Osaka University Graduate School of Medicine, Suita, Osaka, 565-0871 Japan

** JPHC Study Group members are listed at the following site:
http://epi.ncc.go.jp/en/jphc/781/3838.html

Supplemental Table 1. Baseline characteristics of eligible participants according to the final inclusion status

Characteristics	Included in the final analysis	Excluded from the final analysis
**Number of participants**	74,530	20,762
Female (%)	54.4	45.1
Age at baseline (years), mean ±SE[2]	51.6 ±0.03	53.7 ±0.06
Body mass index (kg/m^2), mean ±SE[2]	23.5 ±0.01	23.6 ±0.02
Current smoker (%)	26.4	32.6
Current drinker (%)	46.6	48.9
Sports or physical exercise almost daily (%)	4.8	6.0
History of hypertension (%)	16.0	21.2
History of diabetes (%)	4.1	7.0
Attended health examination or screening within the past 1 year (%)	78.8	77.4

[1] Of those who returned the completed questionnaires, 22,762 participants were excluded due to self-reported history of cancer, stroke, or myocardial infarction at baseline; missing information on variables required for propensity score matching; having a stroke before cancer diagnosis; having a cancer diagnosis after the censor date; or exclusion after propensity score matching due to censoring before the index date.

[2] Standard Error.

Supplemental Table 2. Hazard ratios of stroke during follow-up by stroke type after one-to-one matching without replacement

	All Stroke			Cerebral Infarction			Intracerebral Hemorrhage		
	Subjects without cancer	Cancer survivors	95% CI	Subjects without cancer	Cancer survivors	95% CI	Subjects without cancer	Cancer survivors	95% CI
Person-years (n= 557,885)									
All Cancer									
Number of stroke cases	423	279		304	194		92	59	
Adjusted HRs[1]	1.00	1.07	(0.89-1.29)	1.00	1.05	(0.84-1.31)	1.00	1.05	(0.69-1.58)
Gastric Cancer[2]									
Number of stroke cases	95	79		70	54		20	17	
Adjusted HRs[1]	1.00	0.97	(0.68-1.38)	1.00	0.89	(0.58-1.36)	1.00	1.00	(0.46-2.16)
Colorectal Cancer[3]									
Number of stroke cases	80	77		57	59		19	12	
Adjusted HRs[1]	1.00	1.15	(0.8-1.67)	1.00	1.18	(0.77-1.82)	1.00	0.83	(0.36-1.93)
Lung Cancer[4]									
Number of stroke cases	52	25		36	19		15	5	
Adjusted HRs[1]	1.00	1.31	(0.68-2.52)	1.00	1.45	(0.68-3.13)	1.00	0.80	(0.21-2.98)

[1] The number of stroke cases in this table was calculated after propensity score matching with replacement, in which propensity scores were predicted by age, gender, PHC area, smoking status (never, former, <20 cigarettes/day, ≥20 cigarettes/day), BMI (<18.5, 18.5 - <25, 25-<30, 30+), alcohol intake (never/former, <1 time/w, regular (g/d) [<23, 23-<46, 46-<69, 69-<92, 92+]), leisure-time sports or physical exercise (<almost daily, almost daily), history of diabetes or hypertension, and undergoing a health examination (no, yes).

[2] Excluding subjects with incident cancer during follow-up other than gastric cancer.

[3] Excluding subjects with incident cancer during follow-up other than colorectal cancer.

[4] Excluding subjects with incident cancer during follow-up other than lung cancer.

Supplemental Table 3. Hazard ratios of stroke during follow-up by clinical stage at cancer diagnosis

	Subjects without cancer	Localized		Regional		Distant	
		Cancer survivors	95% CI	Cancer survivors	95% CI	Cancer survivors	95% CI
All Cancer							
Number of all stroke cases	1,963	175		57		16	
Adjusted HRs[1]	1.00	0.95	(0.76-1.18)	0.81	(0.54-1.22)	0.85	(0.38-1.90)
Gastric Cancer[2]							
Number of all stroke cases	2,056	56		15		6	
Adjusted HRs[1]	1.00	1.06	(0.79-1.43)	1.29	(0.71-2.36)	**4.60**	**(1.73-12.24)**
Colorectal Cancer[3]							
Number of all stroke cases	1,975	53		14		4	
Adjusted HRs[1]	1.00	1.09	(0.81-1.46)	0.94	(0.53-1.66)	2.02	(0.72-5.66)
All Cancer							
Number of cerebral infarction cases	1,227	130		36		11	
Adjusted HRs[1]	1.00	1.03	(0.80-1.33)	0.68	(0.41-1.13)	0.89	(0.35-2.28)
Gastric Cancer[2]							
Number of cerebral infarction cases	1,262	39		8		6	
Adjusted HRs[1]	1.00	1.22	(0.85-1.74)	0.94	(0.41-2.19)	**6.17**	**(2.21-17.27)**
Colorectal Cancer[3]							
Number of cerebral infarction cases	1,235	40		12		3	
Adjusted HRs[1]	1.00	1.25	(0.89-1.77)	1.18	(0.65-2.17)	2.16	(0.65-7.13)

[1] The number of stroke cases in this table was calculated after propensity score matching with replacement, in which propensity scores were predicted by age, gender, PHC area, smoking status (never, former, <20 cigarettes/day, ≥20 cigarettes/day), BMI (<18.5, 18.5 - <25, 25-<30, 30+), alcohol intake (never/former, <1 time/w, regular (g/d) [<23, 23-<46, 46-<69, 69-<92, 92+]), leisure-time sports or physical exercise (<almost daily, almost daily), history of diabetes or hypertension, and undergoing a health examination (yes, no).

[2] Excluding subjects with incident cancer during follow-up other than gastric cancer.

[3] Excluding subjects with incident cancer during follow-up other than colorectal cancer.
